# Supplementary figures and images for: Modeling susceptibility to drug-induced long QT with a panel of subject-specific induced pluripotent stem cells
Source: eLife. 2017 Jan 30;6:e19406. doi: 10.7554/eLife.19406 (PMC5279943; doi:10.7554/eLife.19406)

P11007

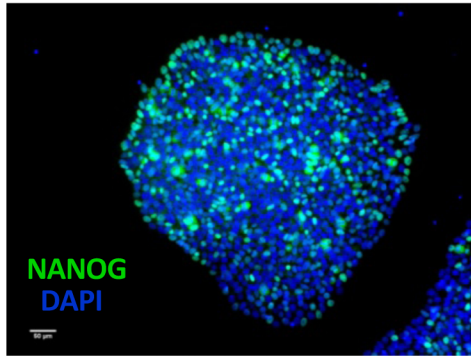

P11008

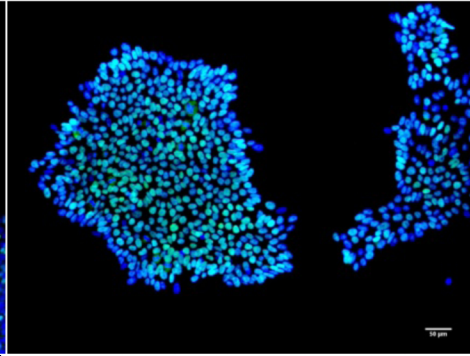

P11009

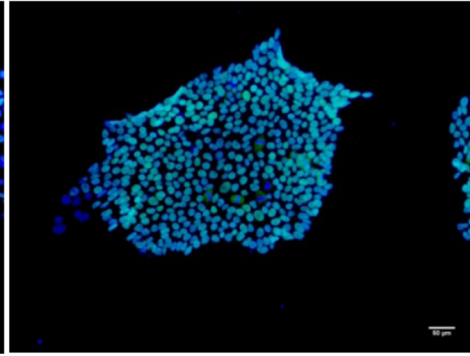

P11013

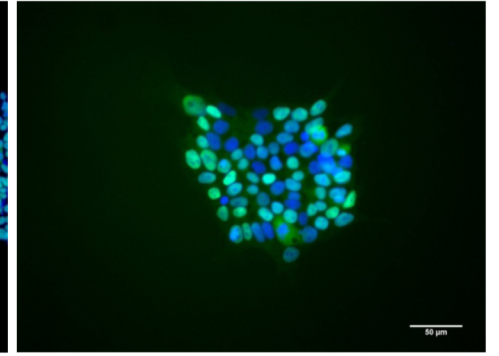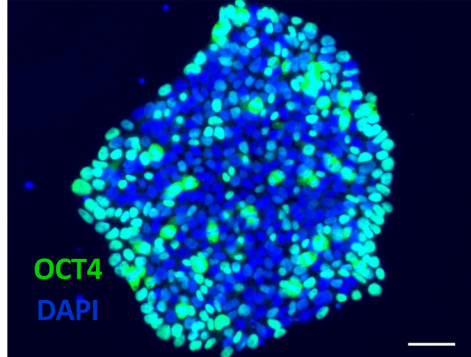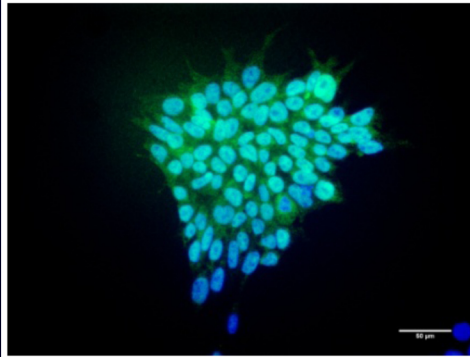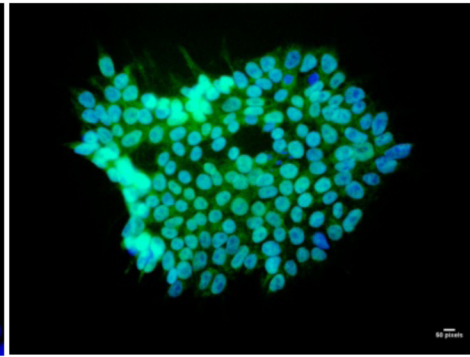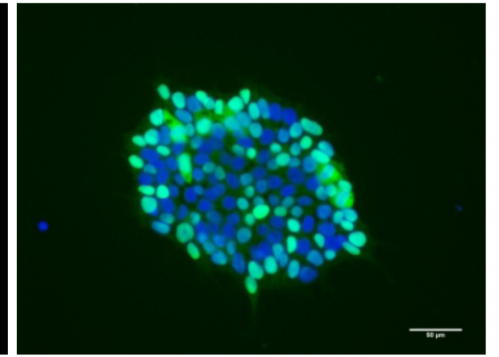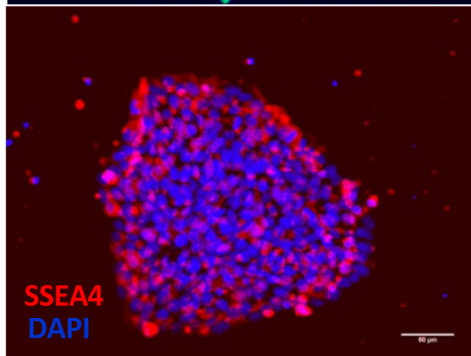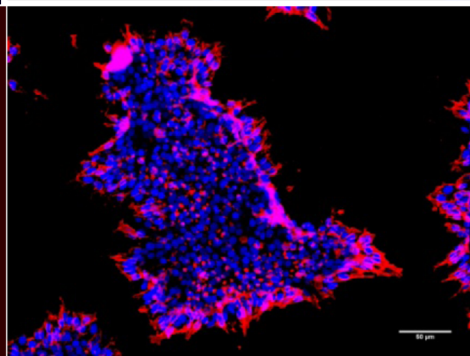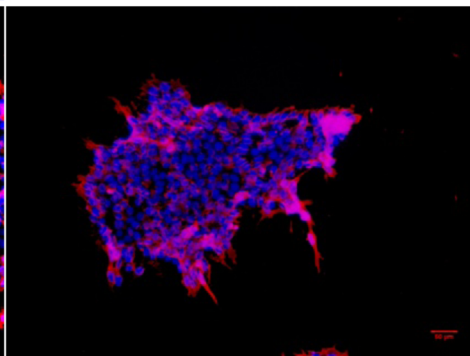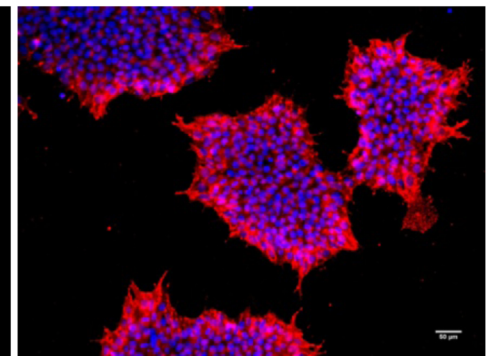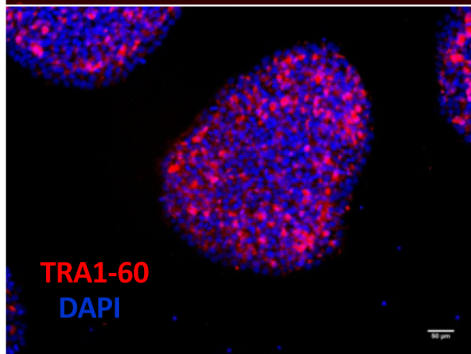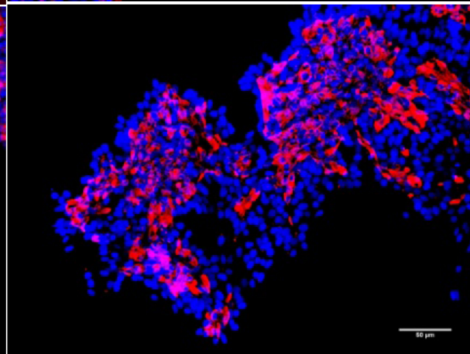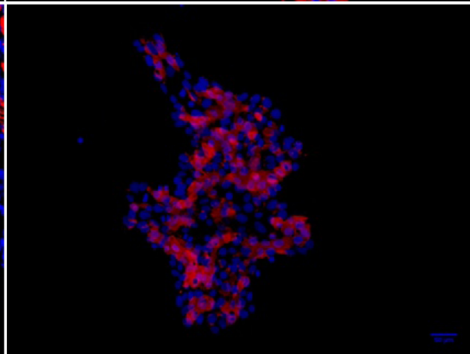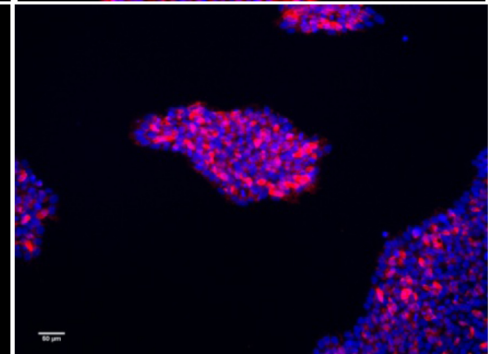

**P11014**

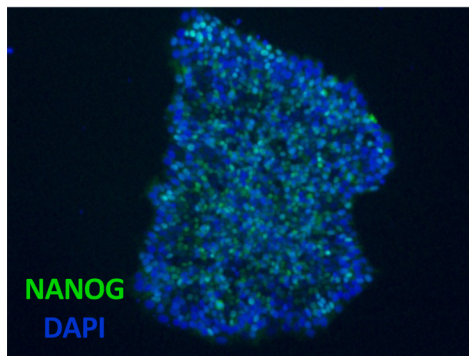

**P11015**

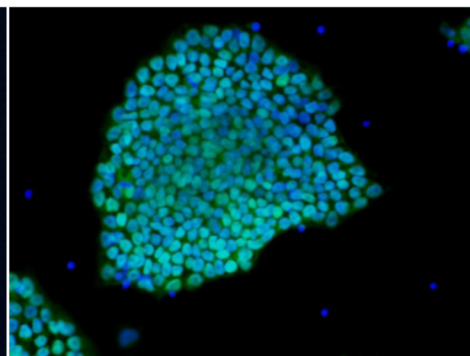

**P11018**

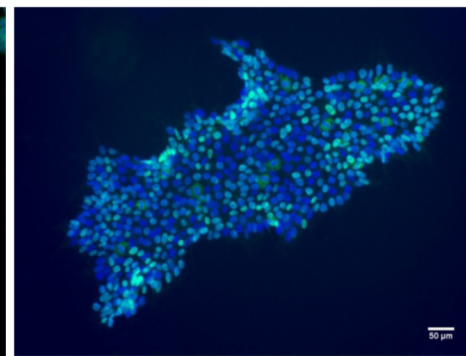

**P11019**

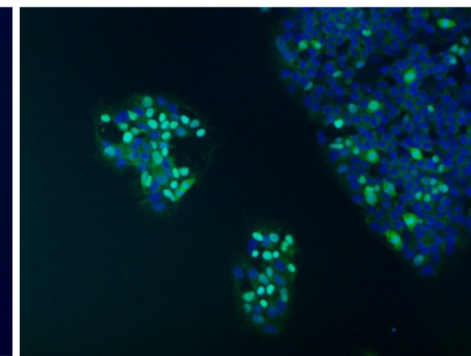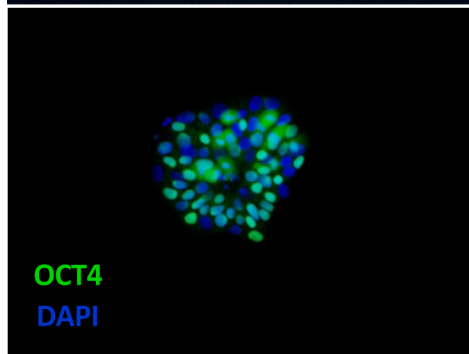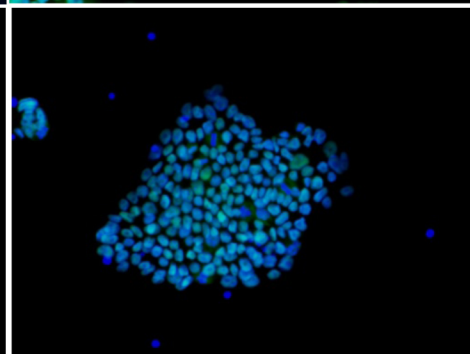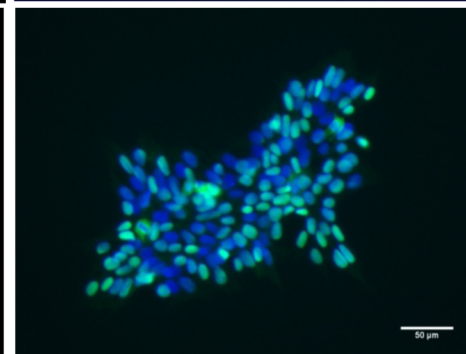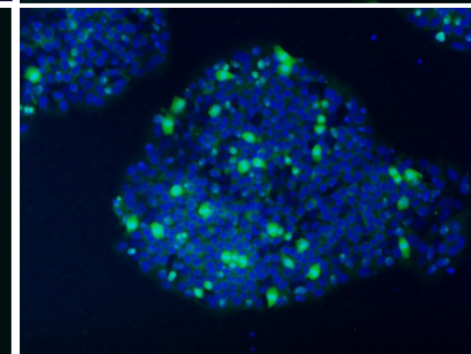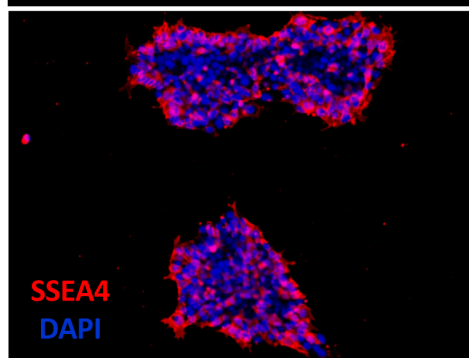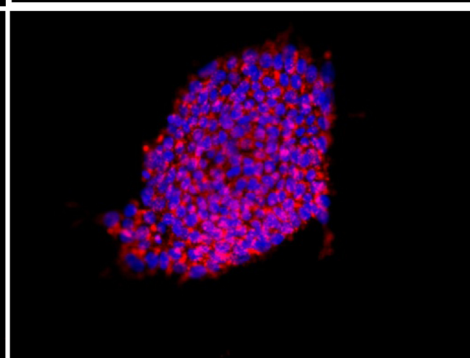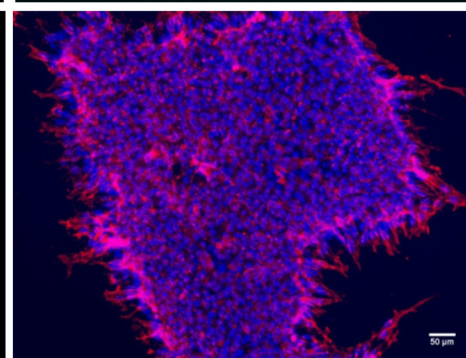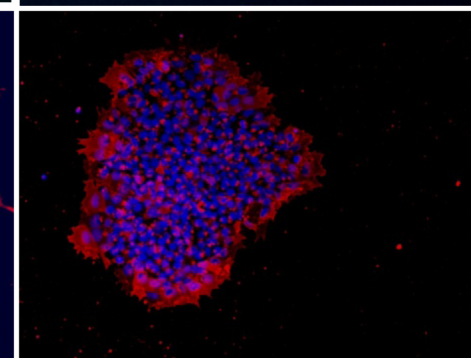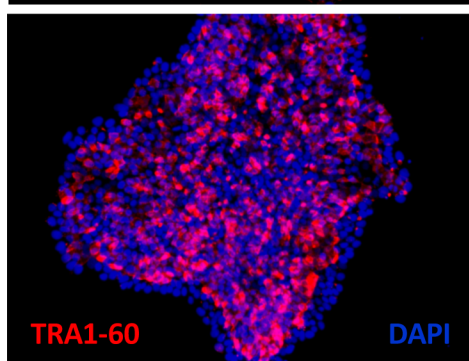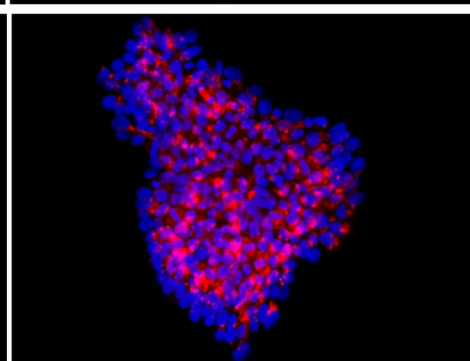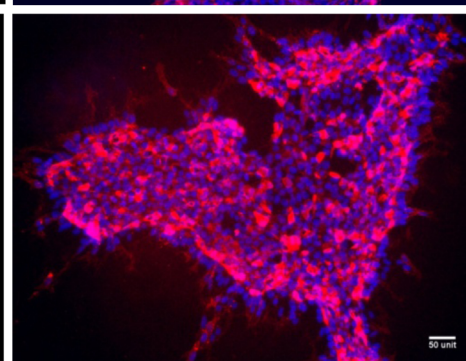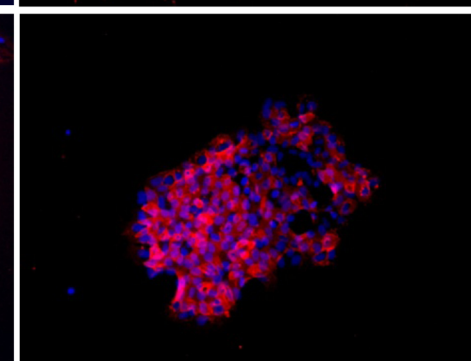

P11020

P11021

P11023

P11024

NANOG  
DAPI

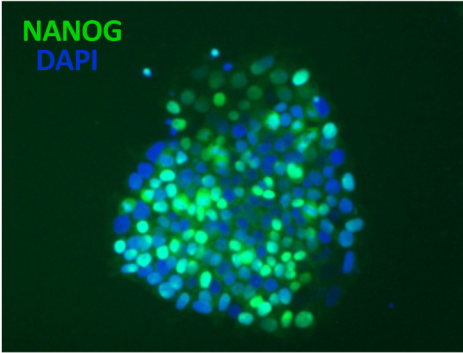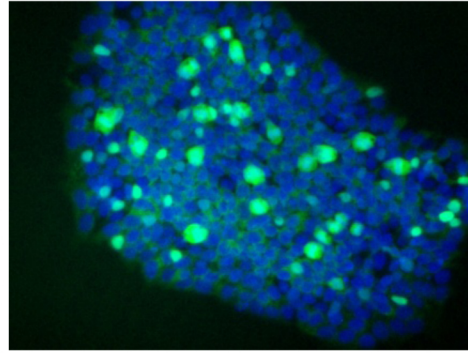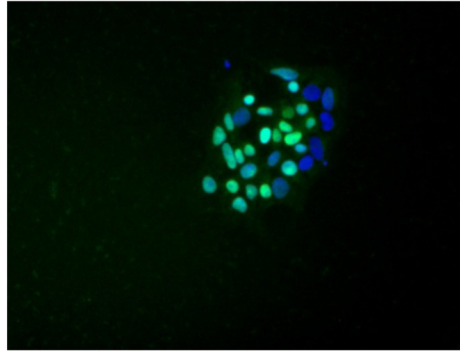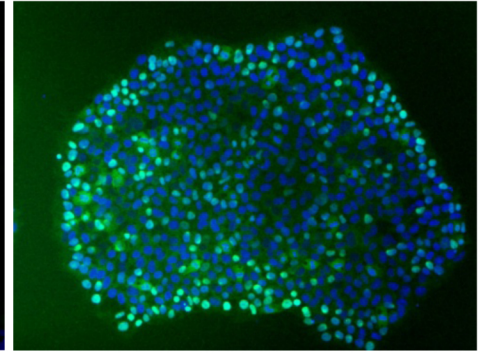

OCT4  
DAPI

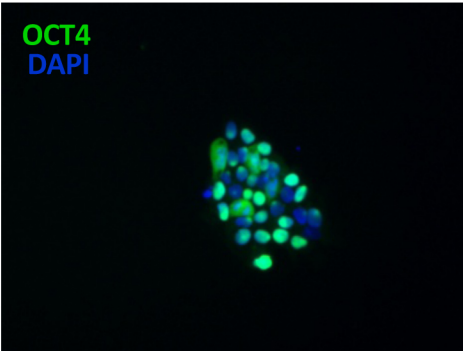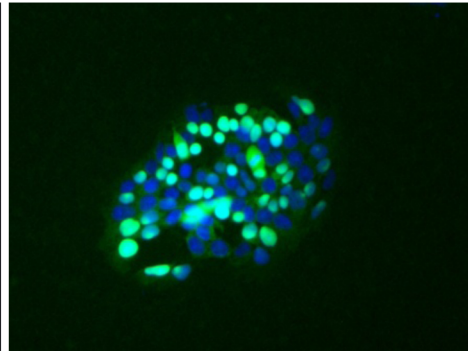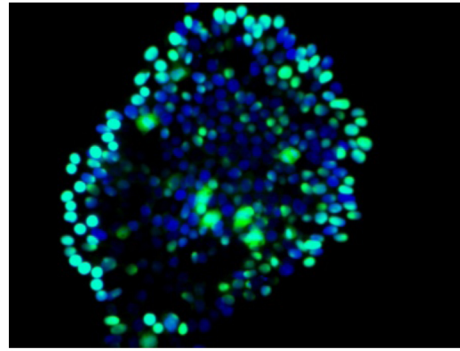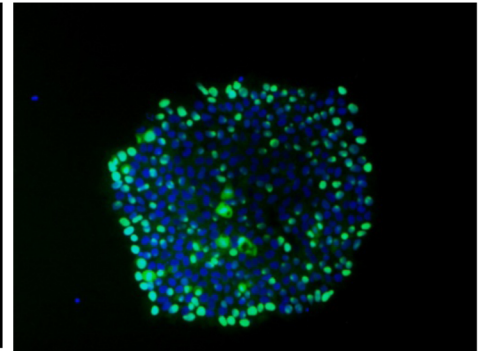

SSEA4  
DAPI

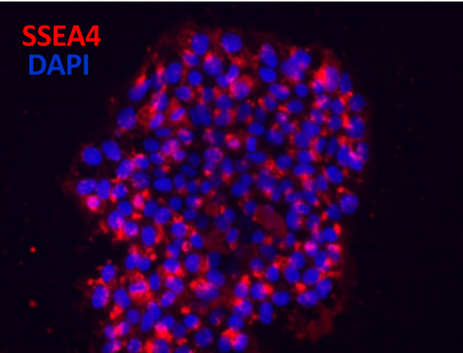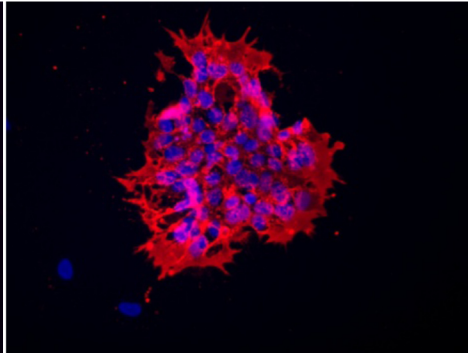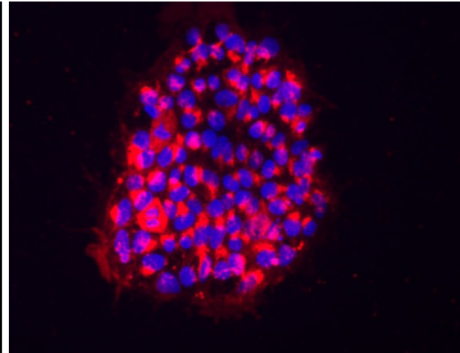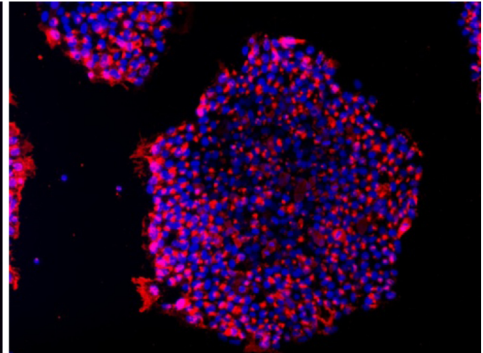

TRA1-60  
DAPI

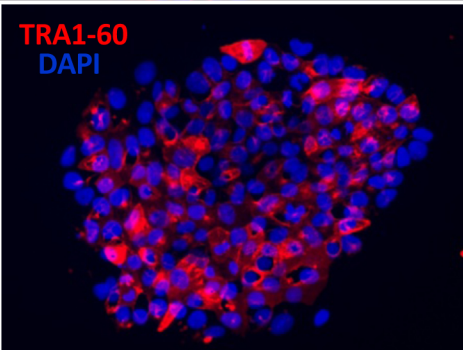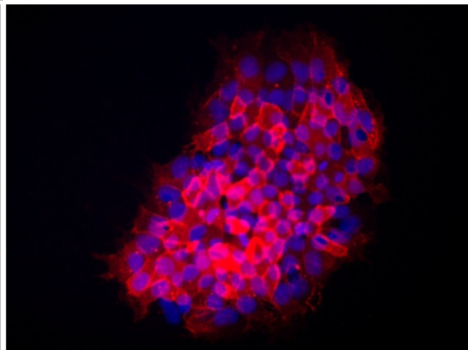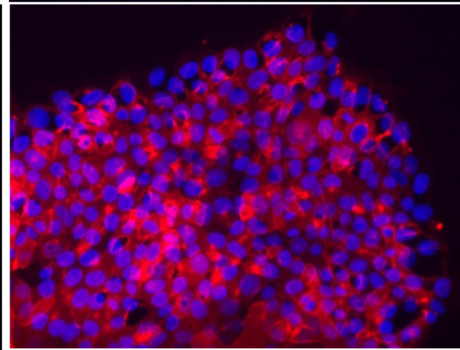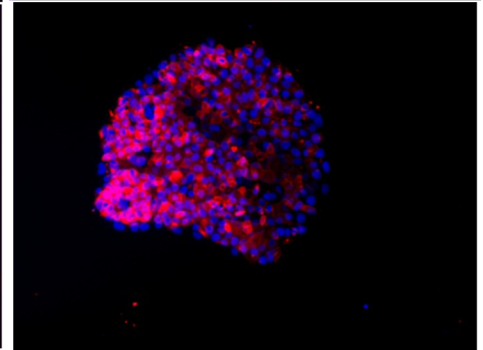

**P11026**

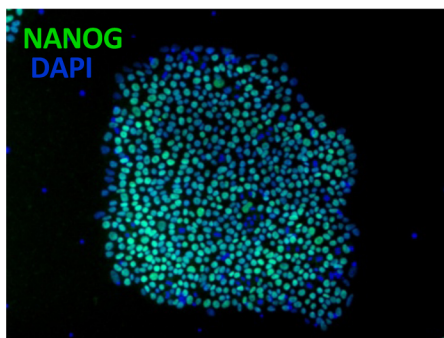

**P11028**

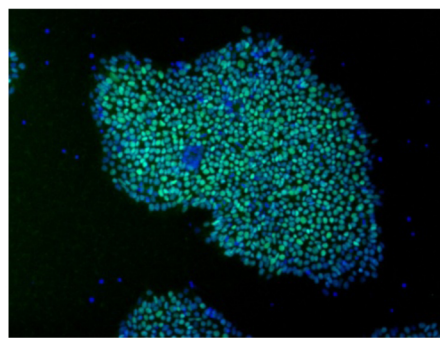

**P11029**

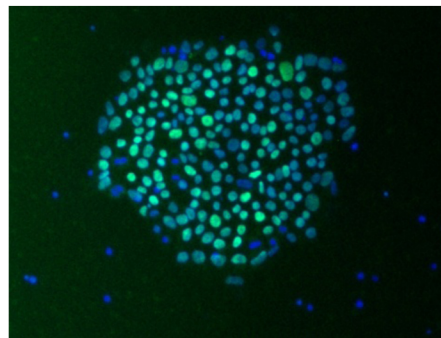

**P11030**

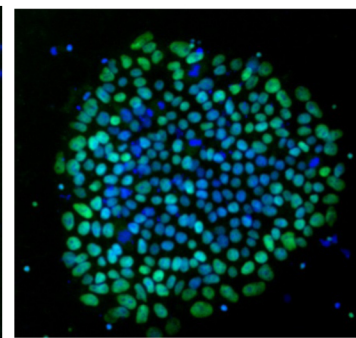

OCT4  
DAPI

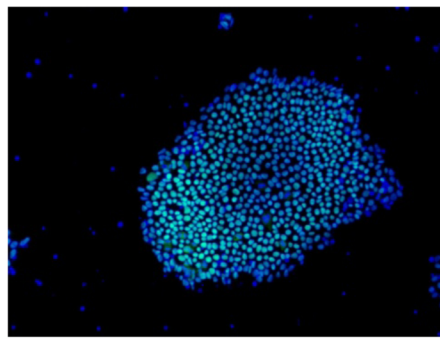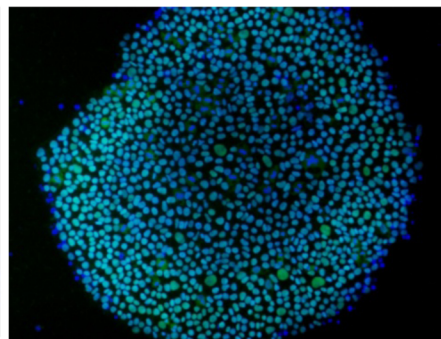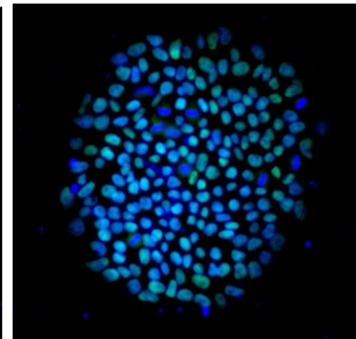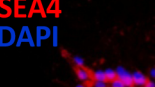

**SSEA4**  
**DAPI**

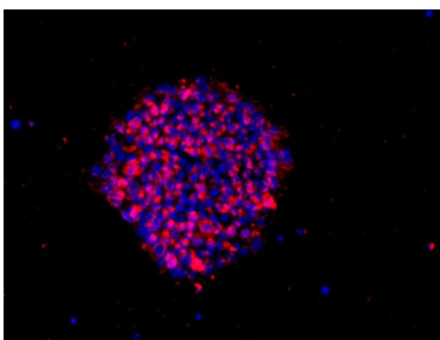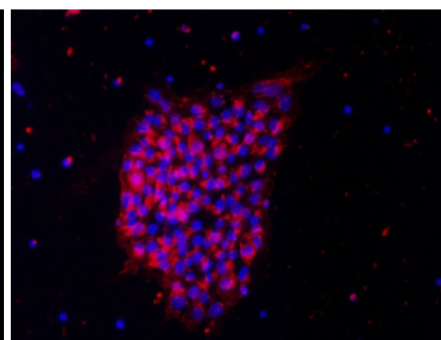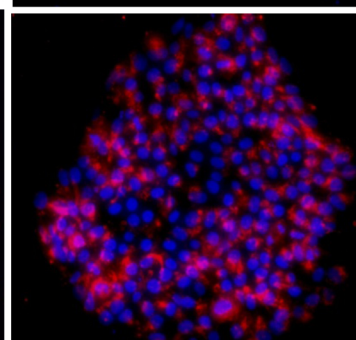

TRA1-60  
DAPI

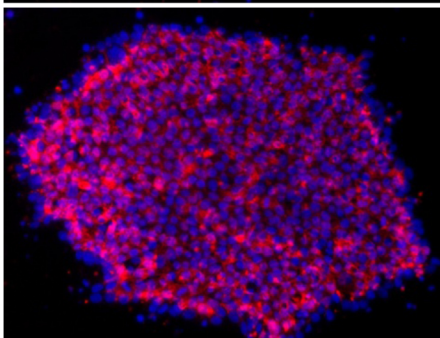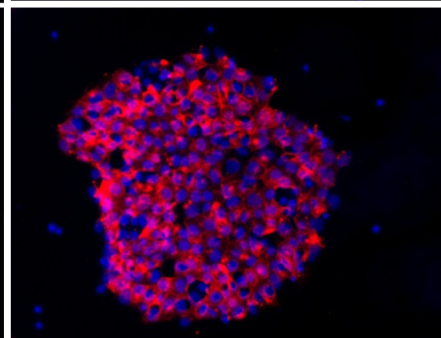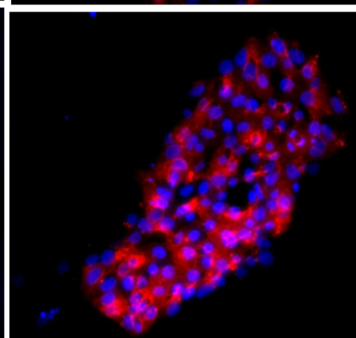

P11031

HESCs

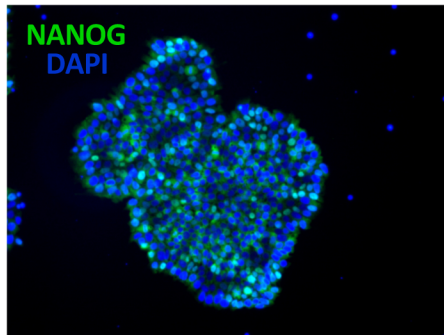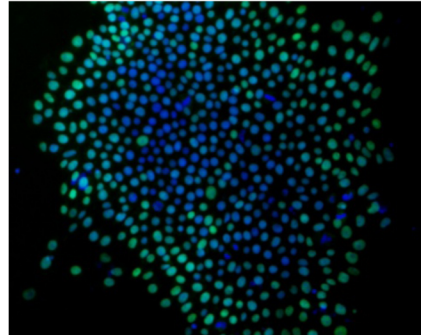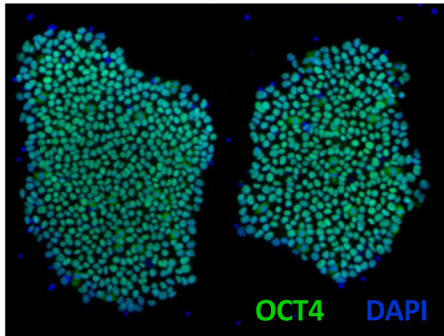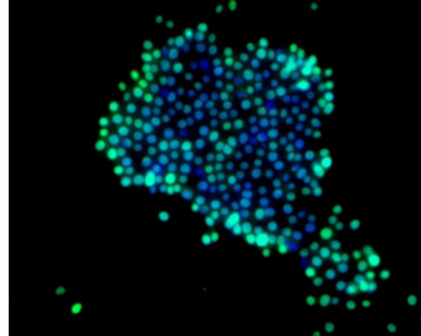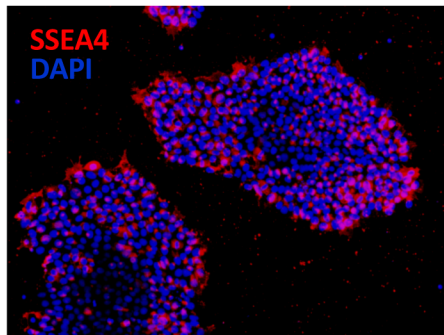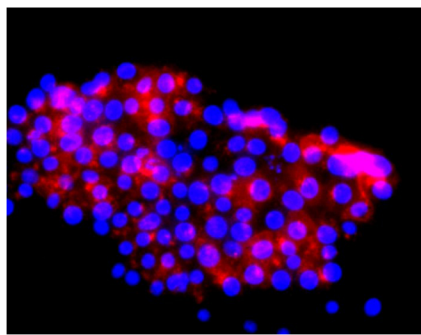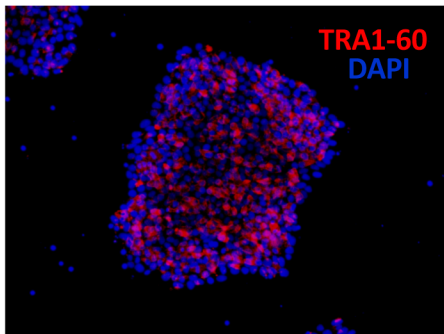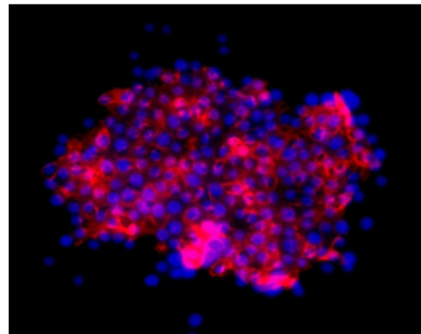

Supplement: Figure 2—source data 1. — Representative immunostaining for a panel of pluripotent stem cell markers, including Nanog, OCT4, SSEA4 and TRA1-60. Results are reported for each cell line. Typical staining in hESC is reported as a positive control. Scale bar 50 μM. DOI: http://dx.doi.org/10.7554/eLife.19406.008 [file elife-19406-fig2-data1.pdf]

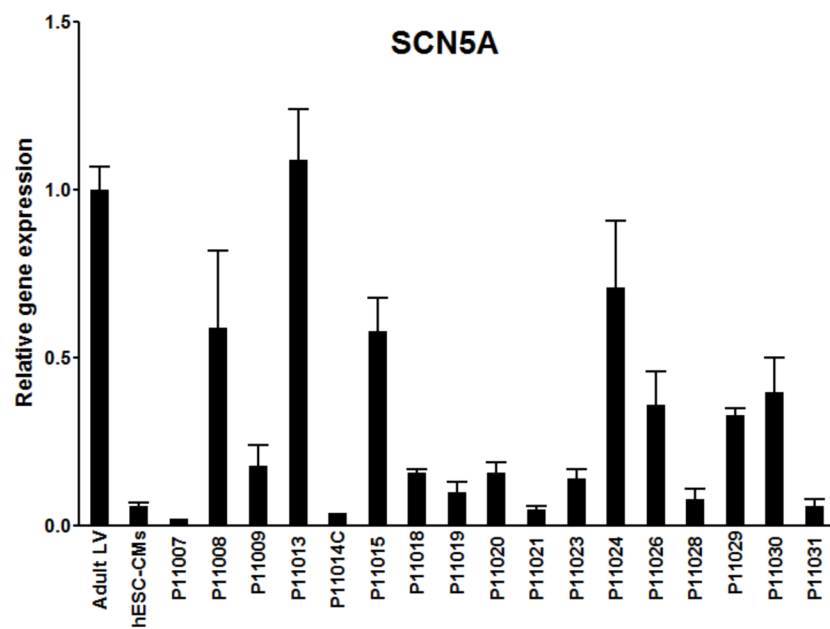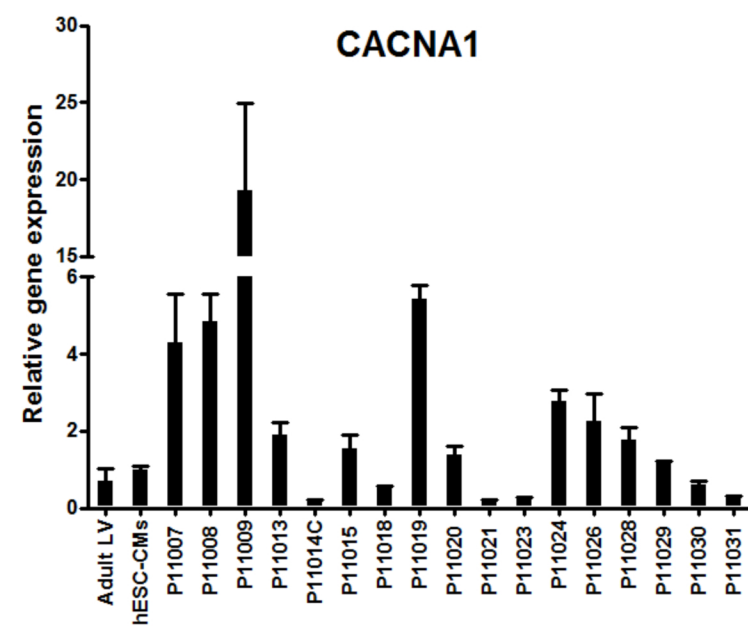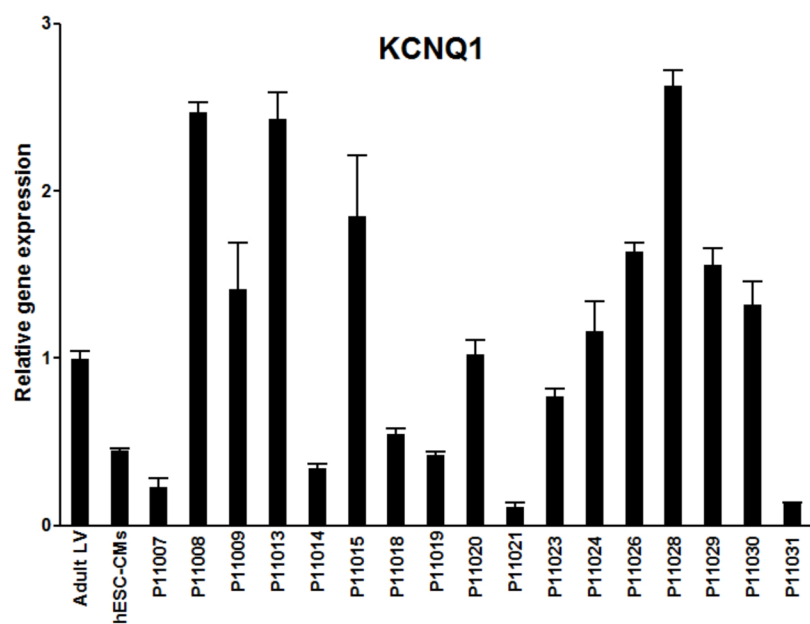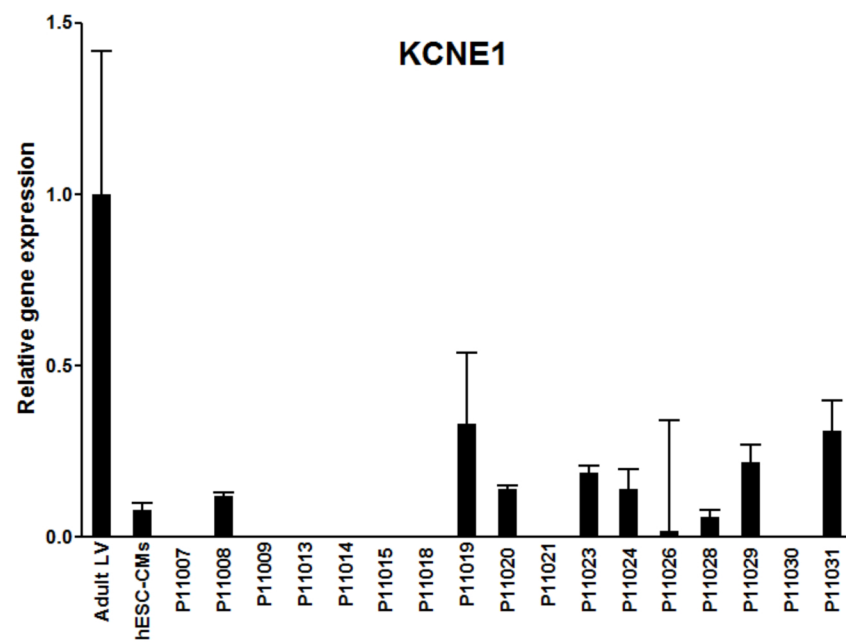

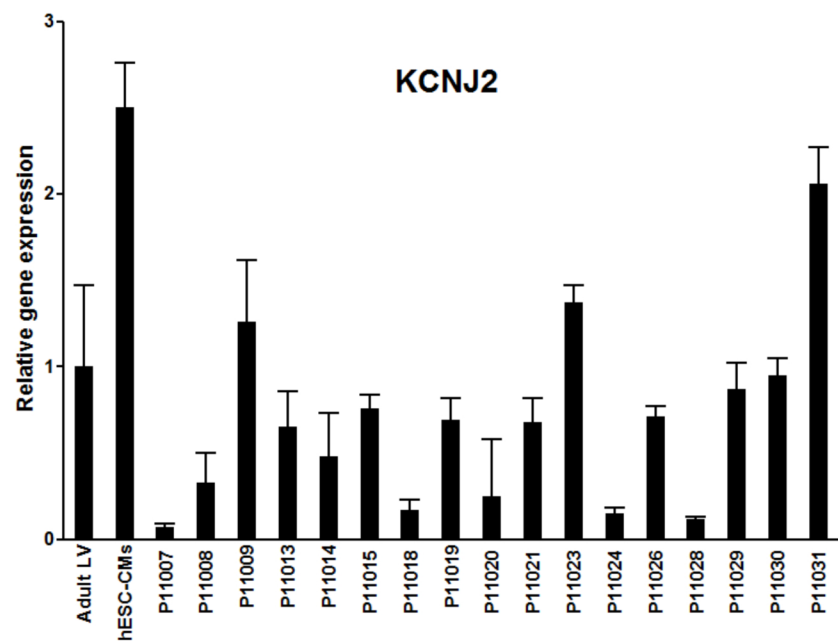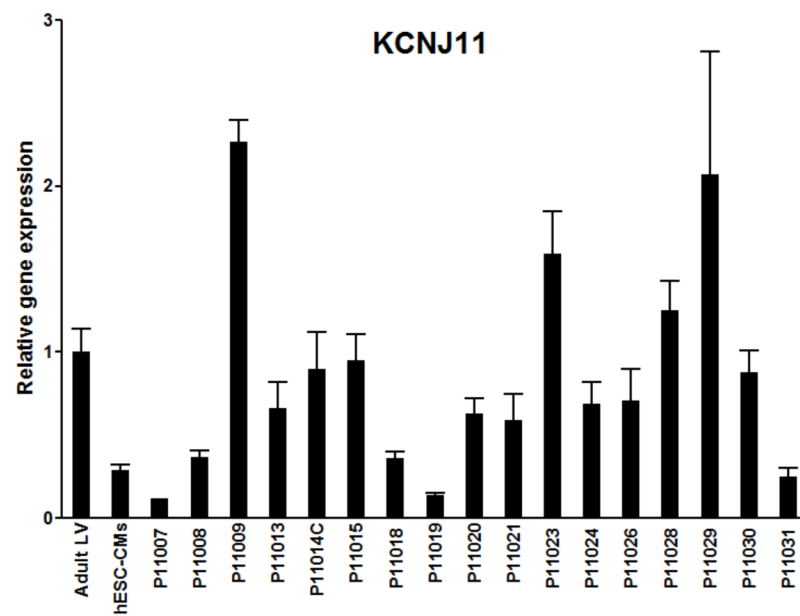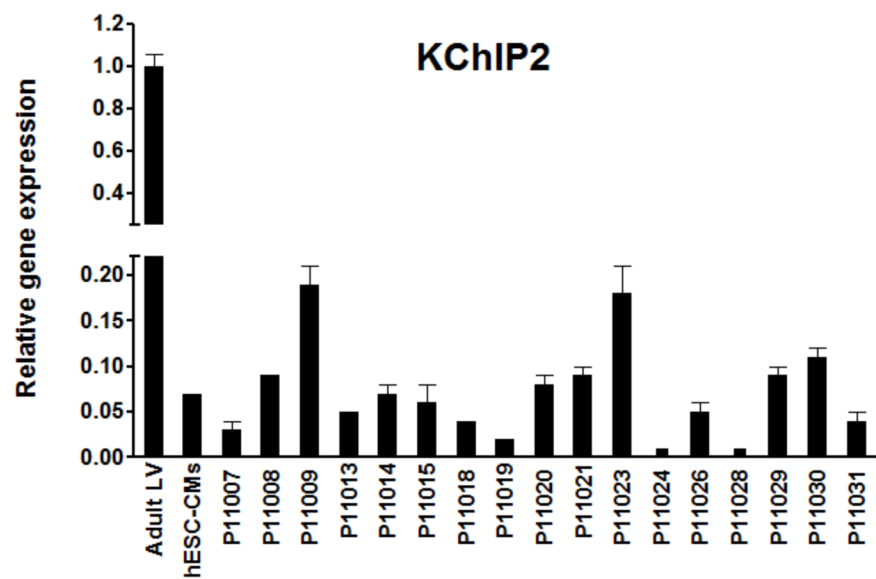

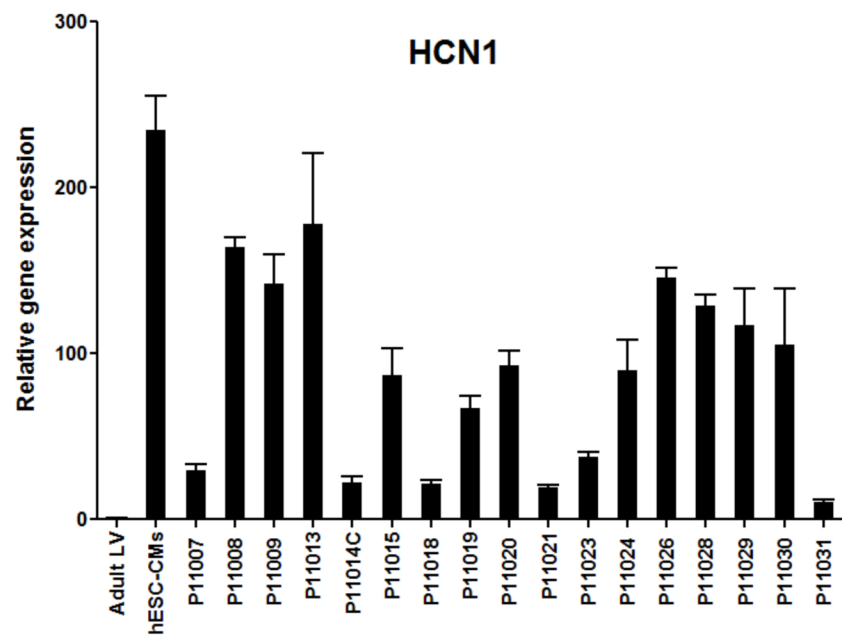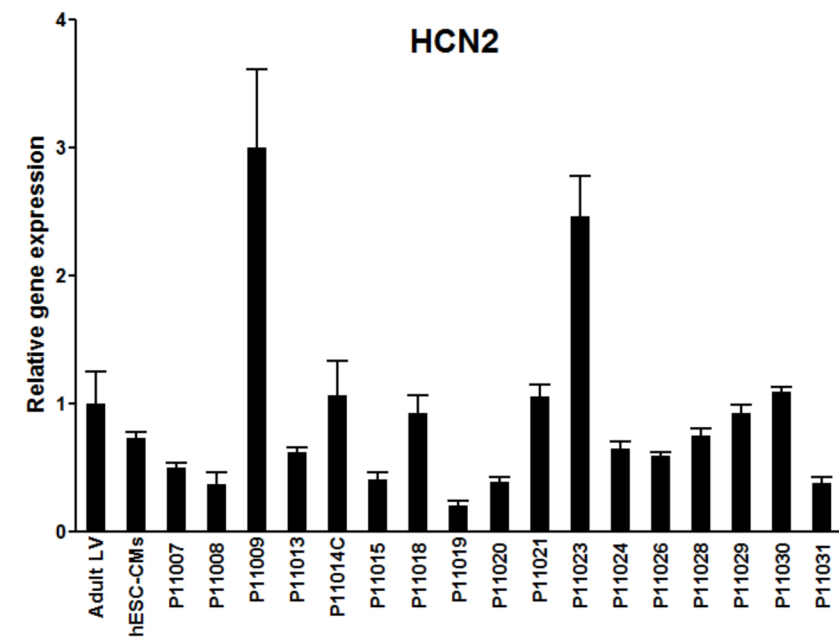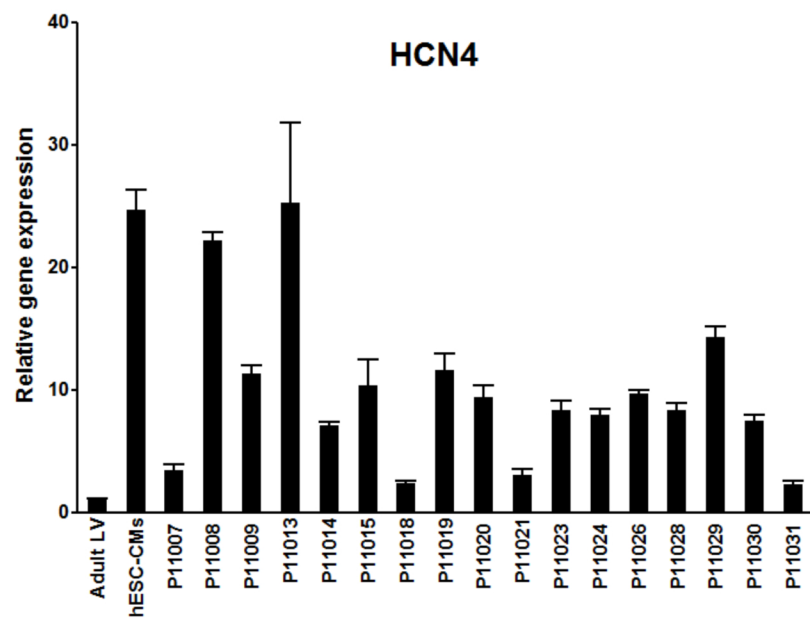

Supplement: Figure 2—source data 2. — Quantitative PCR results for the expression of 10 major cardiac ion channels including SCN5A, CACNA1, KCNQ1, KCNE1, KCNJ2, KCNJ11, KChIP2, HCN1, HCN2, HCN4. KCND3 was not detected. Adult LV tissue is used as a positive control and the level of expression in human embryonic stem cells-derived cardiomyocytes as a comparator. DOI: http://dx.doi.org/10.7554/eLife.19406.009 [file elife-19406-fig2-data2.pdf]
